# Crystal structure of 7-bromo-4-oxo-4*H*-chromene-3-carbaldehyde

**DOI:** 10.1107/S1600536814018108

**Published:** 2014-08-13

**Authors:** Yoshinobu Ishikawa

**Affiliations:** a School of Pharmaceutical Sciences, University of Shizuoka, 52-1 Yada, Suruga-ku, Shizuoka 422-8526, Japan

**Keywords:** crystal structure, chromone, C—H⋯O hydrogen bonding, stacking inter­action

## Abstract

In the title compound, C_10_H_5_BrO_3_, a brominated 3-formyl­chromone derivative, all atoms are essentially coplanar (r.m.s. = 0.0631 Å for the non-H atoms), with the largest deviation from the least-squares plane [0.215 (3) Å] being for the formyl O atom. In the crystal, mol­ecules are linked into tapes through C—H⋯O hydrogen bonds and these tapes are assembled by stacking inter­actions [centroid–centroid distance between the pyran rings = 3.858 (3) Å] to form supra­molecular layers that stack along the *c* axis.

## Related literature   

For related structures, see: Ishikawa (2014*a* ▶,*b* ▶). For halogen bonding, see: Auffinger *et al.* (2004 ▶); Metrangolo *et al.* (2005 ▶); Wilcken *et al.* (2013 ▶); Sirimulla *et al.* (2013 ▶). For halogen–halogen inter­actions, see: Metrangolo & Resnati (2014 ▶); Mukherjee & Desiraju (2014 ▶).
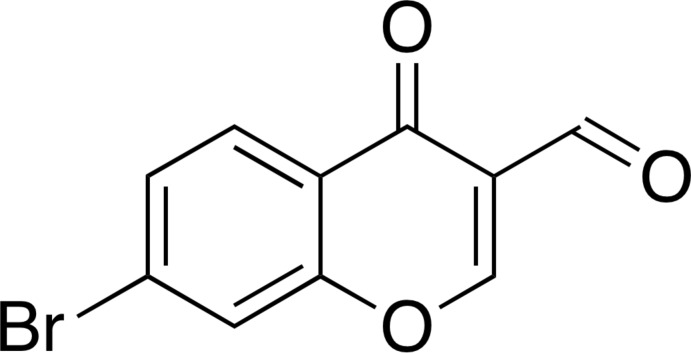


## Experimental   

### Crystal data  

C_10_H_5_BrO_3_
*M* *_r_* = 253.05
Monoclinic, 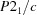

*a* = 3.8580 (18) Å
*b* = 6.054 (4) Å
*c* = 37.268 (13) Å
β = 90.39 (4)°
*V* = 870.4 (8) Å^3^
*Z* = 4
Mo *K*α radiation
μ = 4.71 mm^−1^
*T* = 100 K
0.45 × 0.20 × 0.10 mm

### Data collection  

Rigaku AFC-7R diffractometer
Absorption correction: ψ scan (North *et al.*, 1968 ▶) *T*_min_ = 0.339, *T*_max_ = 0.624
4817 measured reflections
1980 independent reflections
1710 reflections with *F* ^2^ > 2σ(*F* ^2^)
*R*_int_ = 0.024
3 standard reflections every 150 reflections intensity decay: 4.8%

### Refinement  

*R*[*F* ^2^ > 2σ(*F* ^2^)] = 0.038
*wR*(*F* ^2^) = 0.109
*S* = 1.07
1980 reflections
127 parameters
H-atom parameters constrained
Δρ_max_ = 1.26 e Å^−3^
Δρ_min_ = −1.73 e Å^−3^

### 

Data collection: *WinAFC Diffractometer Control Software* (Rigaku, 1999 ▶); cell refinement: *WinAFC Diffractometer Control Software*; data reduction: *WinAFC Diffractometer Control Software*; program(s) used to solve structure: *SIR2008* (Burla *et al.*, 2007 ▶); program(s) used to refine structure: *SHELXL97* (Sheldrick, 2008 ▶); molecular graphics: *CrystalStructure* (Rigaku, 2010 ▶); software used to prepare material for publication: *CrystalStructure*.

## Supplementary Material

Crystal structure: contains datablock(s) General, I. DOI: 10.1107/S1600536814018108/tk5337sup1.cif

Structure factors: contains datablock(s) I. DOI: 10.1107/S1600536814018108/tk5337Isup2.hkl

Supporting information file. DOI: 10.1107/S1600536814018108/tk5337Isup3.cml

**Fig. 1.**
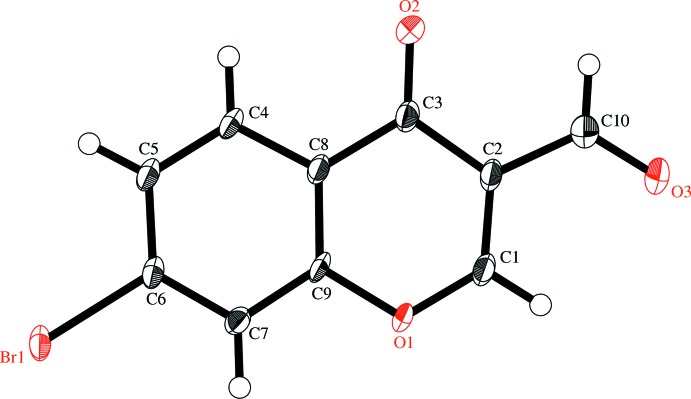
The molecular structure of the title compound with displacement ellipsoids drawn at the 50% probability level. Hydrogen atoms are shown as small spheres of arbitrary radius.

. DOI: 10.1107/S1600536814018108/tk5337fig1.tif
The mol­ecular structure of the title compound with displacement ellipsoids drawn at the 50% probability level. Hydrogen atoms are shown as small spheres of arbitrary radius.

**Fig. 2.**
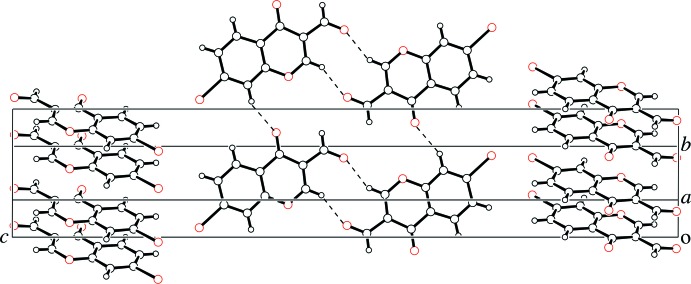
A packing view of the title compound. C—H···O hydrogen bonds are represented by dashed lines.

. DOI: 10.1107/S1600536814018108/tk5337fig2.tif
A packing view of the title compound. C—H⋯O hydrogen bonds are represented by dashed lines.

**Fig. 3.**
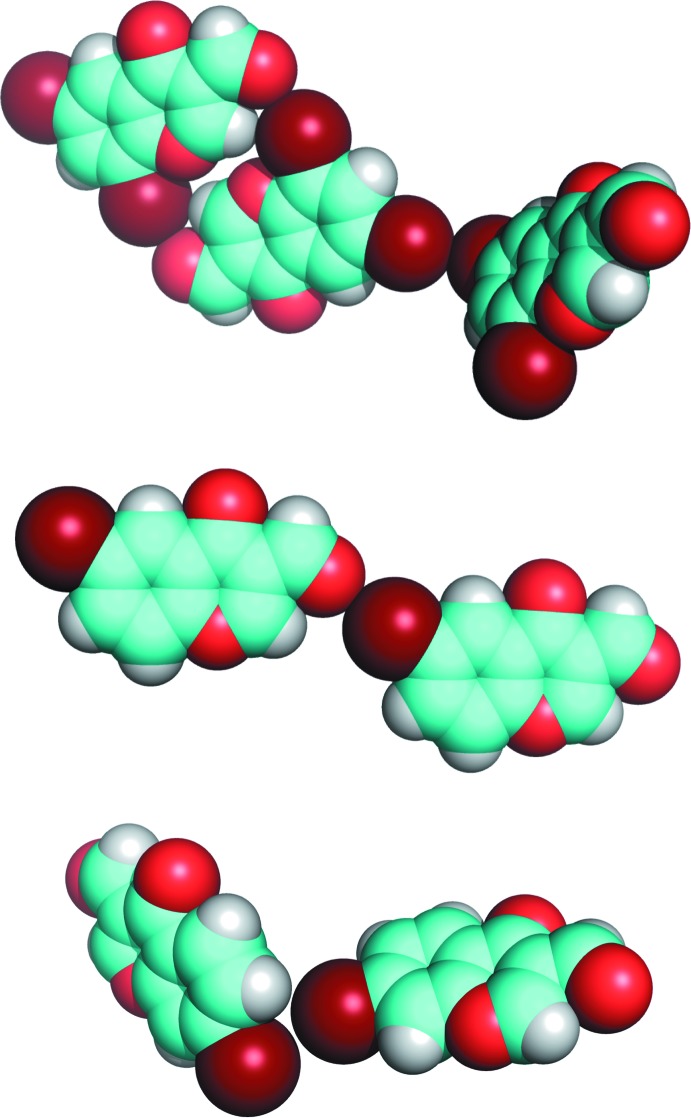
Sphere models of the crystal structures of 6,8-dibromo-4-oxo-4H-chromene-3-carbaldehyde (top, Ishikawa, 2014a), 6-bromo-4-oxo-4H-chromene-3-carbaldehyde (middle, Ishikawa, 2014b), and the title compound (bottom, this work).

H a H b . DOI: 10.1107/S1600536814018108/tk5337fig3.tif
Sphere models of the crystal structures of 6,8-di­bromo-4-oxo-4*H*-chromene-3-carbaldehyde (top, Ishikawa, 2014*a*), 6-bromo-4-oxo-4*H*-chromene-3-carbaldehyde (middle, Ishikawa, 2014*b*), and the title compound (bottom, this work).

CCDC reference: 1018275

Additional supporting information:  crystallographic information; 3D view; checkCIF report

## Figures and Tables

**Table 1:** Hydrogen-bond geometry (Å, °)

*D*—H⋯*A*	*D*—H	H⋯*A*	*D*⋯*A*	*D*—H⋯*A*
C7^i^—H4^i^⋯O2	0.95	2.30	3.149 (4)	149 (1)
C1^ii^—H1^ii^⋯O3	0.95	2.37	3.228 (5)	149 (1)

Symmetry codes: (i) 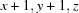; (ii) 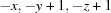.

## supplementary crystallographic information

### S1. Structural commentary

Halogen bonding and halogen···halogen inter­action have recently attracted much attention in medicinal chemistry, chemical biology, supra­molecular chemistry and crystal engineering (Auffinger *et al.*, 2004, Metrangolo *et al*., 2005, Wilcken *et al.*, 2013, Sirimulla *et al.*, 2013, Mukherjee & Desiraju, 2014, Metrangolo & Resnati, 2014). We have recently reported the crystal structures of a dibrominated 3-formyl­chromone derivative 6,8-di­bromo-4-oxo-4*H*-chromene-3-carbaldehyde (Ishikawa, 2014*a*) and a monobrominated 3-formyl­chromone derivative 6-bromo-4-oxo-4*H*-chromene-3-carbaldehyde (Ishikawa, 2014*b*). Halogen bonding between the formyl oxygen atom and the bromine atom at 8-position and type II halogen···halogen inter­action between the bromine atoms at 6-position are observed in 6,8-di­bromo-4-oxo-4*H*-chromene-3-carbaldehyde (Fig.3, top). On the other hand, halogen bonding between the formyl oxygen atom and the bromine atom at 6-position is found in 6-bromo-4-oxo-4*H*-chromene-3-carbaldehyde (Fig.3, middle). As part of our inter­est in these types of chemical bonding, we herein report the crystal structure of a monobrominated 3-formyl­chromone derivative 7-bromo-4-oxo-4*H*-chromene-3-carbaldehyde.

The objective of this study is to reveal whether a short contact is found for the bromine atom at 7-position. The mean deviation of the least-square planes for the non-hydrogen atoms is 0.0631 Å, and the largest deviation is 0.215 (3) Å for the formyl O3 atom (Fig. 1). In the crystal, the molecules are linked through C–H···O hydrogen bonds between the translation-symmetry^i^ and inversion-symmetry equivalents^ii,iii^ to form tapes [i: *x* + 1, *y* + 1, *z*, ii: –*x*, –*y* + 1, –*z* + 1, iii: –*x* + 1, –*y* + 2, –*z* + 1], which are further assembled by stacking inter­actions [centroid–centroid distance between the pyran rings of the 4*H*-chromene units = 3.858 (3) Å], as shown in Fig. 2. A short contact for the bromine atom at 7-position is not observed (Fig. 3, bottom).

### S2. Synthesis and crystallization

To a solution of 4-bromo-2-hy­droxy­aceto­phenone (4.7 mmol) in *N*,*N*-di­methyl­formamide (15 ml) was added drop-wise POCl_3_ (11.6 mmol) at 0 °C. After the mixture was stirred for 14 h at room temperature, water (50 ml) was added. The precipitates were collected, washed with water, and dried *in vacuo* (yield: 84%). ^1^H NMR (400 MHz, CDCl_3_): *δ* = 7.48 (d, 1H, *J* = 8.8 Hz), 7.57 (s, 1H), 8.24 (d, 1H, *J* = 8.8 Hz), 8.52 (s, 1H), 10.37 (s, 1H). DART-MS calcd for [C_10_H_5_BrO_3_ + H^+^]: 252.950, found 252.981. Single crystals suitable for X-ray diffraction were obtained from a 1,2-di­chloro­ethane/cyclo­hexane solution of the title compound at room temperature.

### S3. Refinement

Crystal data, data collection and structure refinement details are summarized in Table 1. The C(*sp*^2^)-bound hydrogen atoms were placed in geometrical positions [C–H 0.95 Å, *U*_iso_(H) = 1.2*U*_eq_(C)], and refined using a riding model.

### Crystal data

**Table:**

C_10_H_5_BrO_3_	*F*(000) = 496.00
*M**_r_* = 253.05	*D*_x_ = 1.931 Mg m^−^^3^
Monoclinic, *P*2_1_/*c*	Mo *K*α radiation, λ = 0.71069 Å
Hall symbol: -P 2ybc	Cell parameters from 25 reflections
*a* = 3.8580 (18) Å	θ = 15.3–17.5°
*b* = 6.054 (4) Å	µ = 4.71 mm^−^^1^
*c* = 37.268 (13) Å	*T* = 100 K
β = 90.39 (4)°	Plate, colourless
*V* = 870.4 (8) Å^3^	0.45 × 0.20 × 0.10 mm
*Z* = 4	

### Data collection

**Table:**

Rigaku AFC-7R diffractometer	*R*_int_ = 0.024
ω scans	θ_max_ = 27.5°
Absorption correction: ψ scan (North *et al.*, 1968)	*h* = −5→2
*T*_min_ = 0.339, *T*_max_ = 0.624	*k* = −7→7
4817 measured reflections	*l* = −48→48
1980 independent reflections	3 standard reflections every 150 reflections
1710 reflections with *F*^2^ > 2σ(*F*^2^)	intensity decay: 4.8%

### Refinement

**Table:**

Refinement on *F*^2^	Secondary atom site location: difference Fourier map
*R*[*F*^2^ > 2σ(*F*^2^)] = 0.038	Hydrogen site location: inferred from neighbouring sites
*wR*(*F*^2^) = 0.109	H-atom parameters constrained
*S* = 1.07	*w* = 1/[σ^2^(*F*_o_^2^) + (0.0756*P*)^2^ + 0.989*P*] where *P* = (*F*_o_^2^ + 2*F*_c_^2^)/3
1980 reflections	(Δ/σ)_max_ = 0.002
127 parameters	Δρ_max_ = 1.26 e Å^−^^3^
0 restraints	Δρ_min_ = −1.73 e Å^−^^3^
Primary atom site location: structure-invariant direct methods	

### Special details

**Table:**

Refinement. Refinement was performed using all reflections. The weighted *R*-factor (*wR*) and goodness of fit (*S*) are based on *F*^2^. *R*-factor (gt) are based on *F*. The threshold expression of *F*^2^ > 2.0 σ(*F*^2^) is used only for calculating *R*-factor (gt).

### Fractional atomic coordinates and isotropic or equivalent isotropic displacement parameters (Å^2^)

**Table:**

	*x*	*y*	*z*	*U*_iso_*/*U*_eq_	
Br1	0.08234 (8)	0.06978 (6)	0.719199 (7)	0.01987 (15)	
O1	0.1286 (6)	0.3047 (4)	0.58604 (6)	0.0198 (5)	
O2	0.6540 (7)	0.8813 (4)	0.60418 (6)	0.0253 (6)	
O3	0.2989 (8)	0.7487 (5)	0.50270 (6)	0.0321 (6)	
C1	0.1969 (9)	0.4490 (6)	0.55957 (8)	0.0199 (7)	
C2	0.3614 (9)	0.6437 (6)	0.56382 (8)	0.0190 (7)	
C3	0.4862 (8)	0.7122 (6)	0.59908 (8)	0.0181 (6)	
C4	0.4730 (9)	0.6080 (6)	0.66421 (8)	0.0173 (6)	
C5	0.3830 (9)	0.4628 (6)	0.69123 (8)	0.0187 (7)	
C6	0.2140 (8)	0.2670 (6)	0.68222 (8)	0.0167 (6)	
C7	0.1322 (8)	0.2111 (6)	0.64716 (8)	0.0170 (6)	
C8	0.3930 (9)	0.5598 (5)	0.62828 (8)	0.0169 (7)	
C9	0.2221 (8)	0.3622 (6)	0.62059 (8)	0.0164 (6)	
C10	0.4165 (9)	0.7874 (7)	0.53244 (9)	0.0256 (8)	
H1	0.1233	0.4106	0.5360	0.0238*	
H2	0.5900	0.7415	0.6700	0.0207*	
H3	0.4357	0.4961	0.7156	0.0224*	
H4	0.0197	0.0759	0.6415	0.0203*	
H5	0.5512	0.9174	0.5356	0.0307*	

### Atomic displacement parameters (Å^2^)

**Table:**

	*U*^11^	*U*^22^	*U*^33^	*U*^12^	*U*^13^	*U*^23^
Br1	0.0190 (3)	0.0305 (3)	0.01010 (19)	−0.00138 (11)	0.00019 (12)	0.00367 (11)
O1	0.0260 (12)	0.0252 (12)	0.0083 (10)	−0.0074 (10)	−0.0036 (9)	−0.0010 (9)
O2	0.0336 (14)	0.0259 (12)	0.0162 (11)	−0.0107 (11)	−0.0033 (10)	−0.0010 (10)
O3	0.0437 (16)	0.0392 (15)	0.0133 (12)	−0.0131 (12)	−0.0067 (11)	0.0046 (11)
C1	0.0209 (16)	0.0291 (18)	0.0095 (14)	−0.0034 (13)	−0.0018 (12)	−0.0010 (12)
C2	0.0214 (16)	0.0261 (16)	0.0096 (13)	−0.0028 (13)	−0.0008 (12)	0.0006 (12)
C3	0.0197 (15)	0.0240 (15)	0.0107 (14)	−0.0011 (12)	0.0011 (11)	−0.0015 (12)
C4	0.0175 (15)	0.0245 (16)	0.0098 (14)	−0.0003 (12)	−0.0021 (11)	−0.0049 (12)
C5	0.0192 (16)	0.0268 (17)	0.0099 (14)	−0.0008 (12)	−0.0015 (12)	−0.0030 (12)
C6	0.0149 (14)	0.0260 (16)	0.0091 (13)	0.0001 (12)	−0.0003 (10)	0.0001 (12)
C7	0.0154 (14)	0.0226 (15)	0.0128 (14)	−0.0035 (12)	−0.0017 (11)	−0.0022 (12)
C8	0.0179 (15)	0.0231 (16)	0.0095 (14)	−0.0023 (12)	−0.0013 (12)	−0.0014 (11)
C9	0.0175 (15)	0.0247 (15)	0.0070 (13)	−0.0010 (12)	−0.0022 (11)	−0.0043 (12)
C10	0.0299 (19)	0.0299 (17)	0.0171 (16)	−0.0078 (15)	−0.0006 (14)	0.0021 (14)

### Geometric parameters (Å, º)

**Table:**

Br1—C6	1.895 (4)	C4—C8	1.403 (5)
O1—C1	1.345 (4)	C5—C6	1.393 (5)
O1—C9	1.379 (4)	C6—C7	1.384 (5)
O2—C3	1.226 (4)	C7—C9	1.394 (5)
O3—C10	1.217 (5)	C8—C9	1.395 (5)
C1—C2	1.348 (5)	C1—H1	0.950
C2—C3	1.457 (5)	C4—H2	0.950
C2—C10	1.474 (5)	C5—H3	0.950
C3—C8	1.473 (5)	C7—H4	0.950
C4—C5	1.382 (5)	C10—H5	0.950
			
O1···C3	2.866 (5)	C10···H1	2.5501
O2···C1	3.561 (5)	H1···H5	3.4843
O2···C4	2.873 (4)	H2···H3	2.3357
O2···C10	2.877 (5)	Br1···H2^i^	3.2968
O3···C1	2.820 (4)	Br1···H2^ii^	3.3454
C1···C7	3.578 (5)	Br1···H3^iii^	3.5917
C1···C8	2.748 (5)	Br1···H3^x^	3.1886
C2···C9	2.772 (5)	Br1···H3^xi^	3.0827
C4···C7	2.810 (5)	O1···H5^ii^	3.4250
C5···C9	2.769 (5)	O2···H4^iv^	3.0581
C6···C8	2.771 (5)	O2···H4^v^	2.2984
O1···O2^i^	3.224 (4)	O3···H1^ix^	2.3733
O1···O2^ii^	3.334 (4)	O3···H1^vii^	2.8324
O1···C3^iii^	3.533 (5)	O3···H5^iii^	3.3041
O2···O1^iv^	3.334 (4)	O3···H5^viii^	2.5428
O2···O1^v^	3.224 (4)	C2···H1^vi^	3.4277
O2···C2^vi^	3.440 (5)	C3···H4^iv^	3.2588
O2···C3^vi^	3.376 (5)	C3···H4^v^	3.3965
O2···C7^iv^	3.263 (4)	C4···H2^iii^	3.5093
O2···C7^v^	3.149 (4)	C4···H4^iv^	3.4320
O2···C8^vi^	3.562 (5)	C5···H2^iii^	3.5776
O2···C9^iv^	3.412 (5)	C6···H2^ii^	3.5270
O3···O3^vii^	3.394 (5)	C6···H3^iii^	3.5409
O3···O3^viii^	3.422 (5)	C7···H2^ii^	3.4509
O3···C1^ix^	3.228 (5)	C7···H4^vi^	3.5285
O3···C1^vii^	3.265 (5)	C8···H4^iv^	3.4764
O3···C10^iii^	3.595 (5)	C10···H1^vi^	3.5572
O3···C10^viii^	3.290 (5)	C10···H1^ix^	3.4940
C1···O3^ix^	3.228 (5)	C10···H1^vii^	3.3402
C1···O3^vii^	3.265 (5)	C10···H5^iii^	3.4327
C1···C2^iii^	3.437 (5)	C10···H5^viii^	3.1048
C1···C3^iii^	3.504 (5)	H1···O3^ix^	2.3733
C2···O2^iii^	3.440 (5)	H1···O3^vii^	2.8324
C2···C1^vi^	3.437 (5)	H1···C2^iii^	3.4277
C3···O1^vi^	3.533 (5)	H1···C10^iii^	3.5572
C3···O2^iii^	3.376 (5)	H1···C10^ix^	3.4940
C3···C1^vi^	3.504 (5)	H1···C10^vii^	3.3402
C4···C6^vi^	3.586 (5)	H1···H1^ix^	3.0401
C4···C7^vi^	3.559 (5)	H1···H5^ii^	3.4115
C5···C6^vi^	3.437 (5)	H1···H5^vii^	3.5617
C6···C4^iii^	3.586 (5)	H2···Br1^iv^	3.3454
C6···C5^iii^	3.437 (5)	H2···Br1^v^	3.2968
C7···O2^i^	3.149 (4)	H2···C4^vi^	3.5093
C7···O2^ii^	3.263 (4)	H2···C5^vi^	3.5776
C7···C4^iii^	3.559 (5)	H2···C6^iv^	3.5270
C8···O2^iii^	3.562 (5)	H2···C7^iv^	3.4509
C8···C9^vi^	3.429 (5)	H2···H4^iv^	3.1684
C9···O2^ii^	3.412 (5)	H2···H4^v^	2.8291
C9···C8^iii^	3.429 (5)	H3···Br1^vi^	3.5917
C10···O3^vi^	3.595 (5)	H3···Br1^xii^	3.1886
C10···O3^viii^	3.290 (5)	H3···Br1^xiii^	3.0827
C10···C10^viii^	3.593 (6)	H3···C6^vi^	3.5409
Br1···H3	2.9226	H4···O2^i^	2.2984
Br1···H4	2.9055	H4···O2^ii^	3.0581
O1···H4	2.5249	H4···C3^i^	3.3965
O2···H2	2.6093	H4···C3^ii^	3.2588
O2···H5	2.5934	H4···C4^ii^	3.4320
O3···H1	2.4901	H4···C7^iii^	3.5285
C1···H5	3.2749	H4···C8^ii^	3.4764
C3···H1	3.2825	H4···H2^i^	2.8291
C3···H2	2.6775	H4···H2^ii^	3.1684
C3···H5	2.6858	H5···O1^iv^	3.4250
C5···H4	3.2949	H5···O3^vi^	3.3041
C6···H2	3.2513	H5···O3^viii^	2.5428
C7···H3	3.2879	H5···C10^vi^	3.4327
C8···H3	3.2793	H5···C10^viii^	3.1048
C8···H4	3.3027	H5···H1^iv^	3.4115
C9···H1	3.1864	H5···H1^vii^	3.5617
C9···H2	3.2628	H5···H5^viii^	2.8597
			
C1—O1—C9	118.0 (3)	C4—C8—C9	118.4 (3)
O1—C1—C2	125.2 (3)	O1—C9—C7	115.7 (3)
C1—C2—C3	120.5 (3)	O1—C9—C8	121.9 (3)
C1—C2—C10	119.6 (3)	C7—C9—C8	122.4 (3)
C3—C2—C10	120.0 (3)	O3—C10—C2	123.6 (4)
O2—C3—C2	123.3 (3)	O1—C1—H1	117.410
O2—C3—C8	122.7 (3)	C2—C1—H1	117.401
C2—C3—C8	114.0 (3)	C5—C4—H2	119.709
C5—C4—C8	120.6 (3)	C8—C4—H2	119.732
C4—C5—C6	119.0 (3)	C4—C5—H3	120.507
Br1—C6—C5	119.2 (3)	C6—C5—H3	120.495
Br1—C6—C7	118.2 (3)	C6—C7—H4	121.481
C5—C6—C7	122.6 (3)	C9—C7—H4	121.508
C6—C7—C9	117.0 (3)	O3—C10—H5	118.165
C3—C8—C4	121.4 (3)	C2—C10—H5	118.205
C3—C8—C9	120.2 (3)		
			
C1—O1—C9—C7	177.4 (3)	C8—C4—C5—C6	0.5 (5)
C1—O1—C9—C8	−2.2 (4)	C8—C4—C5—H3	−179.5
C9—O1—C1—C2	2.5 (5)	H2—C4—C5—C6	−179.5
C9—O1—C1—H1	−177.5	H2—C4—C5—H3	0.5
O1—C1—C2—C3	0.9 (5)	H2—C4—C8—C3	−0.0
O1—C1—C2—C10	−179.4 (3)	H2—C4—C8—C9	179.8
H1—C1—C2—C3	−179.1	C4—C5—C6—Br1	−178.8 (3)
H1—C1—C2—C10	0.6	C4—C5—C6—C7	0.1 (5)
C1—C2—C3—O2	175.8 (3)	H3—C5—C6—Br1	1.1
C1—C2—C3—C8	−4.2 (5)	H3—C5—C6—C7	−179.9
C1—C2—C10—O3	5.6 (5)	Br1—C6—C7—C9	178.02 (18)
C1—C2—C10—H5	−174.4	Br1—C6—C7—H4	−2.0
C3—C2—C10—O3	−174.7 (3)	C5—C6—C7—C9	−0.9 (5)
C3—C2—C10—H5	5.3	C5—C6—C7—H4	179.1
C10—C2—C3—O2	−3.9 (5)	C6—C7—C9—O1	−178.3 (3)
C10—C2—C3—C8	176.1 (3)	C6—C7—C9—C8	1.2 (5)
O2—C3—C8—C4	4.2 (5)	H4—C7—C9—O1	1.7
O2—C3—C8—C9	−175.6 (3)	H4—C7—C9—C8	−178.8
C2—C3—C8—C4	−175.8 (3)	C3—C8—C9—O1	−1.4 (5)
C2—C3—C8—C9	4.4 (4)	C3—C8—C9—C7	179.2 (3)
C5—C4—C8—C3	180.0 (3)	C4—C8—C9—O1	178.8 (3)
C5—C4—C8—C9	−0.2 (5)	C4—C8—C9—C7	−0.7 (5)

Symmetry codes: (i) *x*−1, *y*−1, *z*; (ii) *x*, *y*−1, *z*; (iii) *x*−1, *y*, *z*; (iv) *x*, *y*+1, *z*; (v) *x*+1, *y*+1, *z*; (vi) *x*+1, *y*, *z*; (vii) −*x*+1, −*y*+1, −*z*+1; (viii) −*x*+1, −*y*+2, −*z*+1; (ix) −*x*, −*y*+1, −*z*+1; (x) −*x*, *y*−1/2, −*z*+3/2; (xi) −*x*+1, *y*−1/2, −*z*+3/2; (xii) −*x*, *y*+1/2, −*z*+3/2; (xiii) −*x*+1, *y*+1/2, −*z*+3/2.

### Hydrogen-bond geometry (Å, º)

**Table:**

*D*—H···*A*	*D*—H	H···*A*	*D*···*A*	*D*—H···*A*
C7^v^—H4^v^···O2	0.95	2.30	3.149 (4)	149 (1)
C1^ix^—H1^ix^···O3	0.95	2.37	3.228 (5)	149 (1)

Symmetry codes: (v) *x*+1, *y*+1, *z*; (ix) −*x*, −*y*+1, −*z*+1.
