# Supplementary figures and images for: Crystal structure of 7-bromo-4-oxo-4H-chromene-3-carbaldehyde
Source: Acta Crystallogr Sect E Struct Rep Online. 2014 Aug 13;70(Pt 9):o996. doi: 10.1107/S1600536814018108 (PMC4186120; doi:10.1107/S1600536814018108)

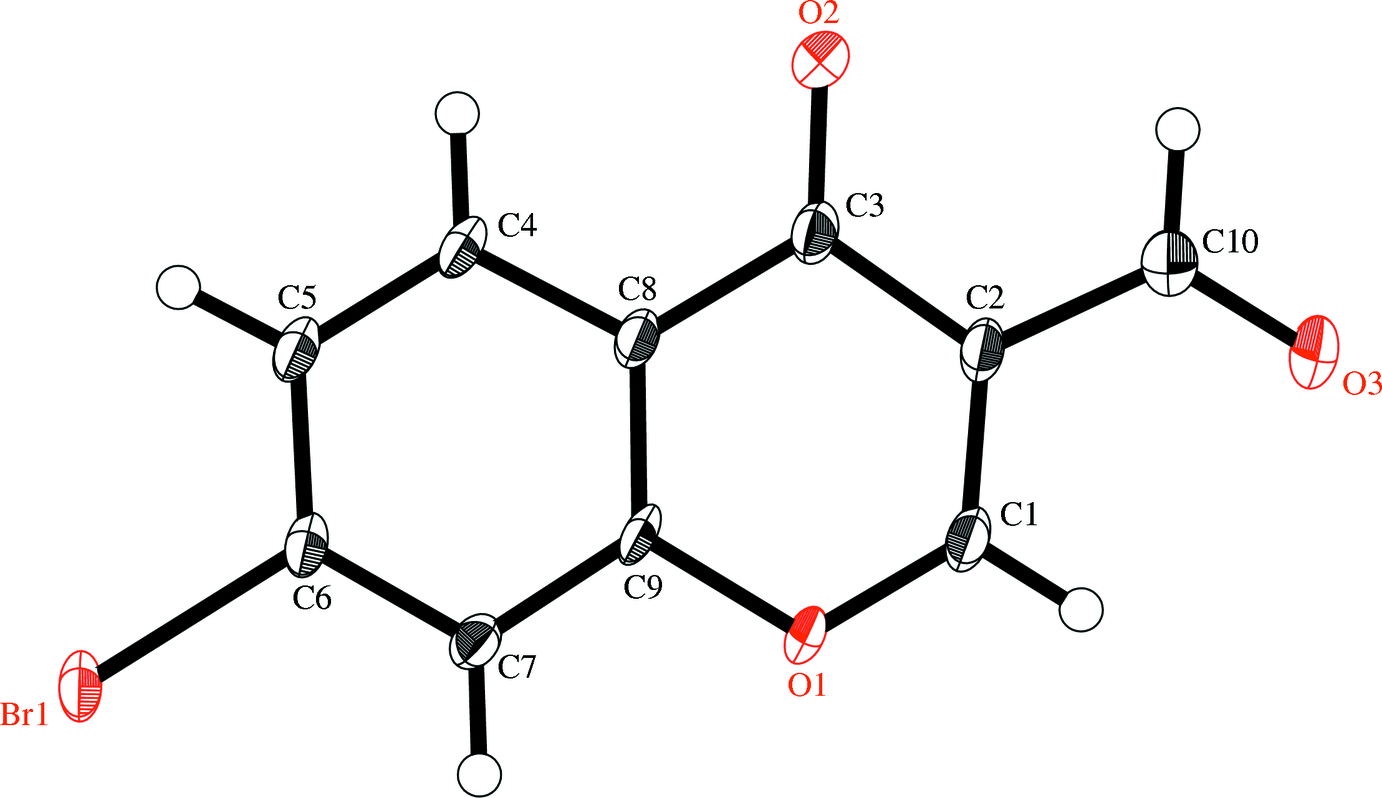

Supplement: Supplementary file 4 [file e-70-0o996-fig1.tif]

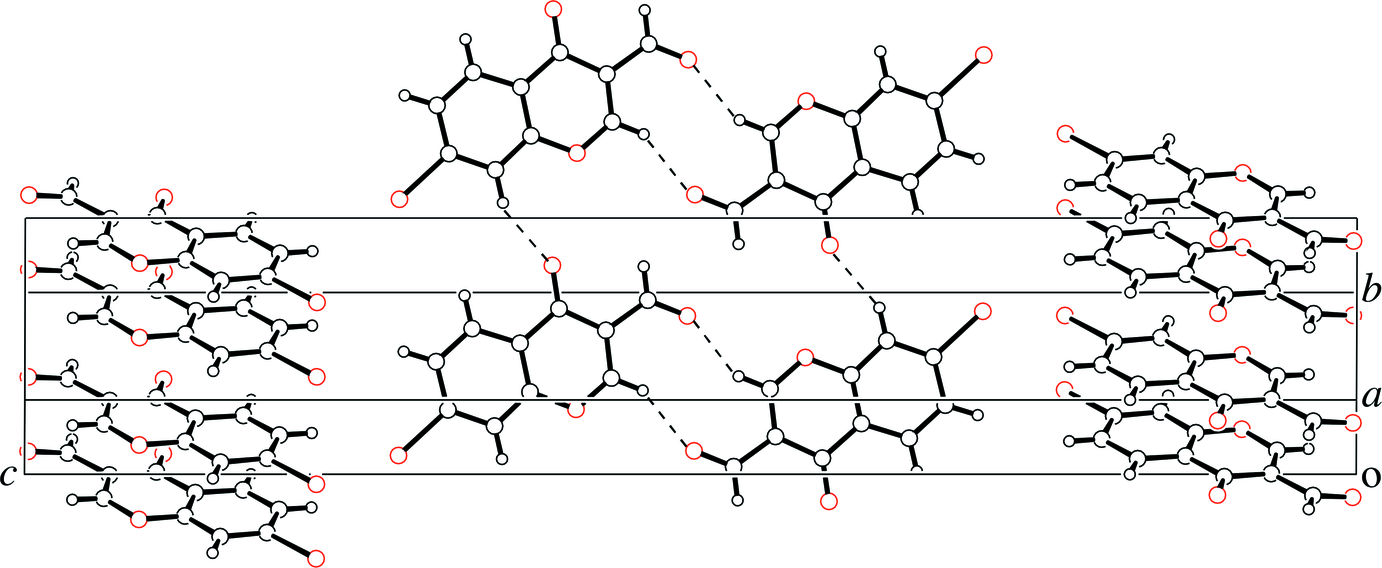

Supplement: Supplementary file 5 [file e-70-0o996-fig2.tif]

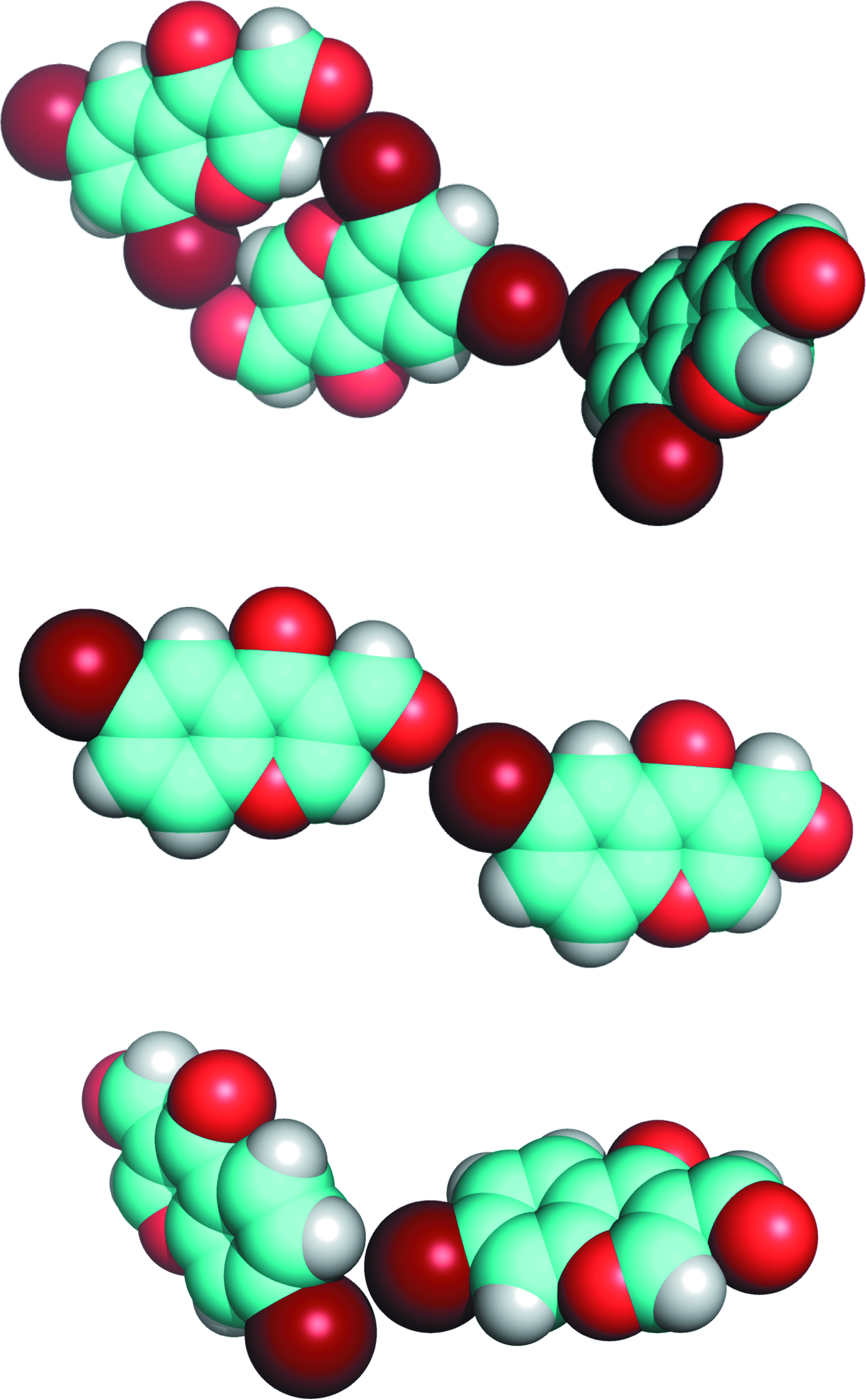

Supplement: Supplementary file 6 [file e-70-0o996-fig3.tif]
